# Longer term follow-up of the randomized phase III trial SWOG S0777: bortezomib, lenalidomide and dexamethasone vs. lenalidomide and dexamethasone in patients (Pts) with previously untreated multiple myeloma without an intent for immediate autologous stem cell transplant (ASCT)

**DOI:** 10.1038/s41408-020-0311-8

**Published:** 2020-05-11

**Authors:** Brian G. M. Durie, Antje Hoering, Rachael Sexton, Muneer H. Abidi, Joshua Epstein, S. Vincent Rajkumar, Angela Dispenzieri, Stephen P. Kahanic, Mohan C. Thakuri, Frederic J. Reu, Christopher M. Reynolds, Robert Z. Orlowski, Bart Barlogie

**Affiliations:** 1 Cedars Sinai Cancer Center, Los Angeles, CA USA; 2 SWOG Statistical Center, Seattle, WA USA; 3 Michigan State University/Spectrum Health Cancer Center, Grand Rapids, MI USA; 4 Myeloma Institute, University of Arkansas for Medical Sciences, Little Rock, AR USA; 5 Division of Hematology, Mayo Clinic, Rochester, MN USA; 6 Sanford NCORP of the North Central Plains/ Siouxland Regional Cancer Center, Sioux City, IA USA; 7 Cancer Care Western NC, Asheville, NC USA; 8 Department of Hematology and Medical Oncology, Taussig Cancer Institute, Cleveland Clinic, Cleveland, OH USA; 9 Michigan Cancer Research Consortium NCORP, St. Joseph Mercy Hospital, Ann Arbor, MI USA; 10 Department of Lymphoma and Myeloma, The University of Texas MD Anderson Cancer Center, Houston, TX USA

**Keywords:** Myeloma, Myeloma

## Abstract

SWOG S0777, a randomized phase III trial, compared bortezomib, lenalidomide and dexamethasone (VRd) with lenalidomide and dexamethasone (Rd). This updated analysis includes 460 patients evaluable for survival endpoints: 225 eligible and analyzable patients were randomized to Rd and 235 to VRd. The 6-month induction was six 28-day cycles of Rd and eight 21-day cycles of VRd followed by Rd maintenance for all patients. Median follow up is 84 months. Median PFS is 41 months for VRd and 29 months for Rd: stratified hazard ratio (96% Wald Confidence Interval) was 0.742 (0.594, 0.928) and one-sided stratified log-rank *P*-value 0.003. Median OS for VRd is still not reached with median OS for Rd being 69 months: stratified hazard ratio (96% Wald Confidence Interval) was 0.709 (0.543, 0.926) and stratified two-sided *P*-value was 0.0114. Both PFS and OS were improved with VRd versus Rd adjusting for age (*P*-values: 0.013 [PFS]; 0.033 [OS])). Median duration of Rd maintenance was 17.1 months. The addition of bortezomib to lenalidomide dexamethasone for induction therapy results in a statistically significant and clinically meaningful improvement in PFS as well as better OS. VRd continues to represent an appropriate standard of care irrespective of age.

## Introduction

The combination of bortezomib, lenalidomide, and dexamethasone was selected at the time of trial design in 2007 to achieve the maximum response in the frontline setting for myeloma therapy. Both bortezomib and lenalidomide were at the time approved for use in the relapsed setting, but still under evaluation in patients with previously untreated multiple myeloma.

Bortezomib is a proteasome inhibitor which decreases proliferation and reverses chemoresistance^1–3^. Lenalidomide is an immunomodulatory agent which exhibits multifaceted, anti-myeloma activity by enhancing immune function, disrupting aberrant stromal cell support as well as having direct anti-myeloma cell effects^4–7^. Both bortezomib and lenalidomide inhibit NF-κB and in combination demonstrate enhanced proapoptotic effects^8,9^. Additionally, dexamethasone further enhances anti-myeloma activity^10,11^. Indeed, prior to the current study, the VRd combination showed promising activity in both the relapsed and newly diagnosed settings^12–14^.

The SWOG S0777 trial was the first phase 3 open-label trial in newly diagnosed patients to evaluate the combination of VRd versus Rd. The results were published in 2017 and demonstrated significantly improved PFS and OS with VRd^15^. The present report outlines the longer-term outcomes with a data cut at May 15, 2018.

## Methods

### Patients and study design

The SWOG S0777 randomized, open-label phase 3 trial was done at Southwest Oncology Group (SWOG) and National Clinical Trials (NCTN) member institutions as listed in the appendix. Patients aged 18 years or older with newly diagnosed myeloma were eligible. Key inclusion criteria were: presence of CRAB criteria (C = calcium elevation; R = renal impairment; A = anemia; B = bone involvement) with measurable disease^16^. No patients with asymptomatic disease were included in this trial. Eastern Cooperative Oncology Group (ECOG) performance status 0–3 was acceptable^17^. Allowable blood count values were: hemoglobin ≥9 g/dL; absolute neutrophil count ≥1 × 10³ cells per mm³; platelet count ≥80,000/mm³. Major exclusion criteria were: creatinine clearance ≤30 mL/min; cardiac status New York Heart Association class III/IV or recent myocardial infarction; active hepatitis B or C or HIV or uncontrolled other infection; previous cancer prior to study registration or enrollment; or poorly controlled diabetes. The study protocol was approved by the institutional review boards of all participating institutions. All patients provided written informed consent. This trial is registered with ClinicalTrials.gov, number NCT00644228.

### Randomization

Patients were randomly assigned (1:1) to receive initial treatment of bortezomib with lenalidomide and dexamethasone (VRd) or lenalidomide and dexamethasone (Rd). We used a dynamic allocation algorithm developed by Pocock and Simon to balance treatment assignment by the stratification factors. The randomization was stratified based on International Staging System stage (I, II, or III) and intent to transplant (yes vs no)^18^. Patients at participating NCTN institutions were randomly assigned upon registration. Randomization procedures were developed and maintained by the SWOG statistics and data management center. There was no masking to treatment interventions.

### Procedures

The VRd regimen was given as eight 21-day cycles. Bortezomib was given at 1·3 mg/m² intravenously on days 1, 4, 8, and 11 combined with 25 mg oral lenalidomide once a day on days 1–14 plus 20 mg oral dexamethasone on days 1, 2, 4, 5, 8, 9, 11, and 12. The Rd regimen was given as six 28-day cycles and consisted of 25 mg oral lenalidomide once a day for days 1–21 plus 40 mg oral dexamethasone on days 1, 8, 15, and 22. The total amount of lenalidomide administered for induction was balanced for each group (VRd: 2800 mg lenalidomide total dose; Rd: 3150 mg total dose). Patients in the VRd group received herpes simplex virus prophylaxis. All patients received 325 mg oral aspirin once a day to reduce the risk of thromboembolic complications. Upon completion of induction, all patients received ongoing maintenance with 25 mg oral lenalidomide once a day for 21 days plus 40 mg oral dexamethasone once a day for days 1, 8, 15, and 22 of each 28-day cycle. Stem-cell collection was allowed for those patients considering future transplant. With dosage adjustments as necessary using slide adjustment scale within the protocol, maintenance was continued until emergence of progressive disease, toxic effects, or patient withdrawal.

### Outcomes

The primary endpoint was progression-free survival from the time of randomization. Secondary endpoints were overall survival, the rate of overall response (partial response or better), safety, and to bank specimens for future translational medicine research. Data were collected and analyzed by the SWOG statistical center team in standard SWOG cooperative group procedural fashion. Treatment response and disease progression were assessed centrally and followed the international uniform response criteria for multiple myeloma^19^. Disease assessments were done at the end of each cycle. After treatment discontinuation because of toxic effects, disease progression, or patient withdrawal, patients were followed up for disease status every 6 months, until death or for a maximum of 6 years after initial randomization. We did fluorescence in-situ hybridization (FISH) analysis of bone marrow cells at trial entry. Preliminary analyses from available data from 316 patients suggested that 33% were deemed high risk by one or more of the high risk features including t(4;14), t(14;16), or chromosome 17 deletion abnormalities. Individual site FISH testing and reports will be further reviewed as part of data assessment in the present study to confirm details, including cell numbers and percentages as well as possible coexistence of high, intermediate, and good risk features. We used standard percentage cutoff values for each type of FISH test abnormality (typically 5%, but ranging from 1·5% to 7·5%). We collected data for adverse events every 3 months while on treatment and again at the end of induction and maintenance treatment. All adverse events were initially graded according to National Cancer Institute Common.

Terminology Criteria for Adverse Events (CTCAE), version 3.0. From April 6, 2011, serious adverse events were graded according to CTCAE version 4.0. An independent data and safety monitoring committee reviewed unblinded safety data twice a year.

### Statistical analysis

The sample size was based on the assumption of an eligible patient accrual rate of 110 patients per year (440 eligible patients over 4 years), a median progression-free survival of about 3 years in the control group, exponential distribution of progression-free survival, and roughly 2.5 years of additional follow up. The study was designed to detect a hazard ratio of 1.5, with ~87% power and an overall study alpha of 0.05. Thus, to allow for an interim analysis, a one-sided 0.02 significance level was used to assess the primary progression-free survival endpoint. The primary endpoint was evaluated with the use of a group-sequential design, with two planned interim analyses at 1/3 and 2/3 of the total number of events. A Haybittle–Peto approach was used for alpha spending and a one-sided alpha of 0.0025 was used for each interim analysis^20,21^. At the final analysis, a one-sided stratified log-rank test was done at the 0.02 significance level for an overall one-sided alpha of 0.025^22^. We compared progression-free survival and overall survival between treatment groups using a log-rank test stratified according to the factors used for randomization^19,23^. Hazard ratios were estimated by means of a stratified Cox proportional-hazards model^24^. The multivariate analysis were done with a model that was not stratified by, rather adjusted for stratification factors, to provide some idea as to how the stratification factors were associated with outcome. We used the Kolmogorov–Smirnov test to assess assumptions of proportional hazards. There was no evidence of violation of proportional hazards for any of the covariates. Survival curves were based on the Kaplan–Meier method^23^. We compared the overall response rate between groups using a stratified Cochran–Mantel–Haenszel test^25,26^. The odds ratio and corresponding 95% confidence interval were estimated with the use of the Mantel–Haenszel method.

Duration of response was summarized by means of the Kaplan–Meier method. All primary and secondary endpoint analyses were predefined within the protocol. Analyses were done on an intention to treat basis that incorporated all eligible patients. Patients with missing parameters of interest were excluded from multivariate analyses. We used SAS (version 4) for all analyses. Baseline variables were compared using Fisher’s exact test. The safety analysis included all eligible patients who received at least one dose of study treatment and who were evaluated for toxic effects.

### Role of the funding source

The funder agreed to provide support for the study as designed. Funding was provided directly to SWOG with no funding being provided to any individual author. The funder had no role in data collection, data analysis, data interpretation, or writing of the report. The corresponding author had full access to all the data in the study and had final responsibility for the decision to submit for publication.

## Results

Between April 2008 and February 2012, 525 patients at 139 participating SWOG and NCTN institutions were randomly assigned: 264 to VRd and 261 to Rd. As previously reported, the baseline characteristics were well-balanced between treatment groups (see Table 1). Slightly more female patients and those age ≥ 65 years were randomized to the Rd arm.

**Table 1:** Patient characteristics by treatment arm.

Factor	All patients	RD	VRd	*P*-value
Age ≥ 65 yr	197/460 (43%)	106/225 (47%)	91/235 (39%)	0.074
Female	191/460 (42%)	105/225 (47%)	86/235 (37%)	0.030
SWOG performance status > 1	53/441 (12%)	29/216 (13%)	24/225 (11%)	0.384
sb2m ≥ 3.5 mg/L	284/458 (62%)	143/224 (64%)	141/234 (60%)	0.442
CRP ≥ 8 mg/L	125/443 (28%)	65/219 (30%)	60/224 (27%)	0.527
Creatinine ≥ 2 mg/dL	21/460 (5%)	10/225 (4%)	11/235 (5%)	1.000
LDH ≥ 190 U/L	163/454 (36%)	81/223 (36%)	82/231 (35%)	0.922
Albumin < 3.5 g/dL	196/458 (43%)	97/223 (43%)	99/235 (42%)	0.778
Hb < 10 g/dL	147/460 (32%)	70/225 (31%)	77/235 (33%)	0.764
Platelet count < 150 × 10^9^/L	80/460 (17%)	45/225 (20%)	35/235 (15%)	0.176
ISS Stage III	155/460 (34%)	78/225 (35%)	77/235 (33%)	0.694
Intent to transplant	315/460 (68%)	153/225 (68%)	162/235 (69%)	0.841

*n*/*N* (%): *n*—Number with factor, *N*—Number with valid data for factor.

ND: No valid observations for factor.

*P*-values computed using Fisher’s exact test.

*P*-values represent a comparison between groups, not against the overall population.

As a basis for this longer-term follow-up analysis, all data elements were checked and updated with a data cut of May 15, 2018. A full listing of the trial profile with patient distribution throughout the trial is in Appendix 1. For these analyses, for VRd, 235 patients were deemed eligible and analyzable for efficacy with 234 evaluable for toxic effects and 215 assessable for response. For Rd, 225 patients were deemed eligible and analyzable for efficacy with 222 evaluable for toxic effects and 207 assessable for response. At the time of this analysis, 53 patients (12% of eligible patients) are still on maintenance therapy. The median overall follow up was 84 months. The median duration of maintenance was 17.1 months.

**Fig. 1:**
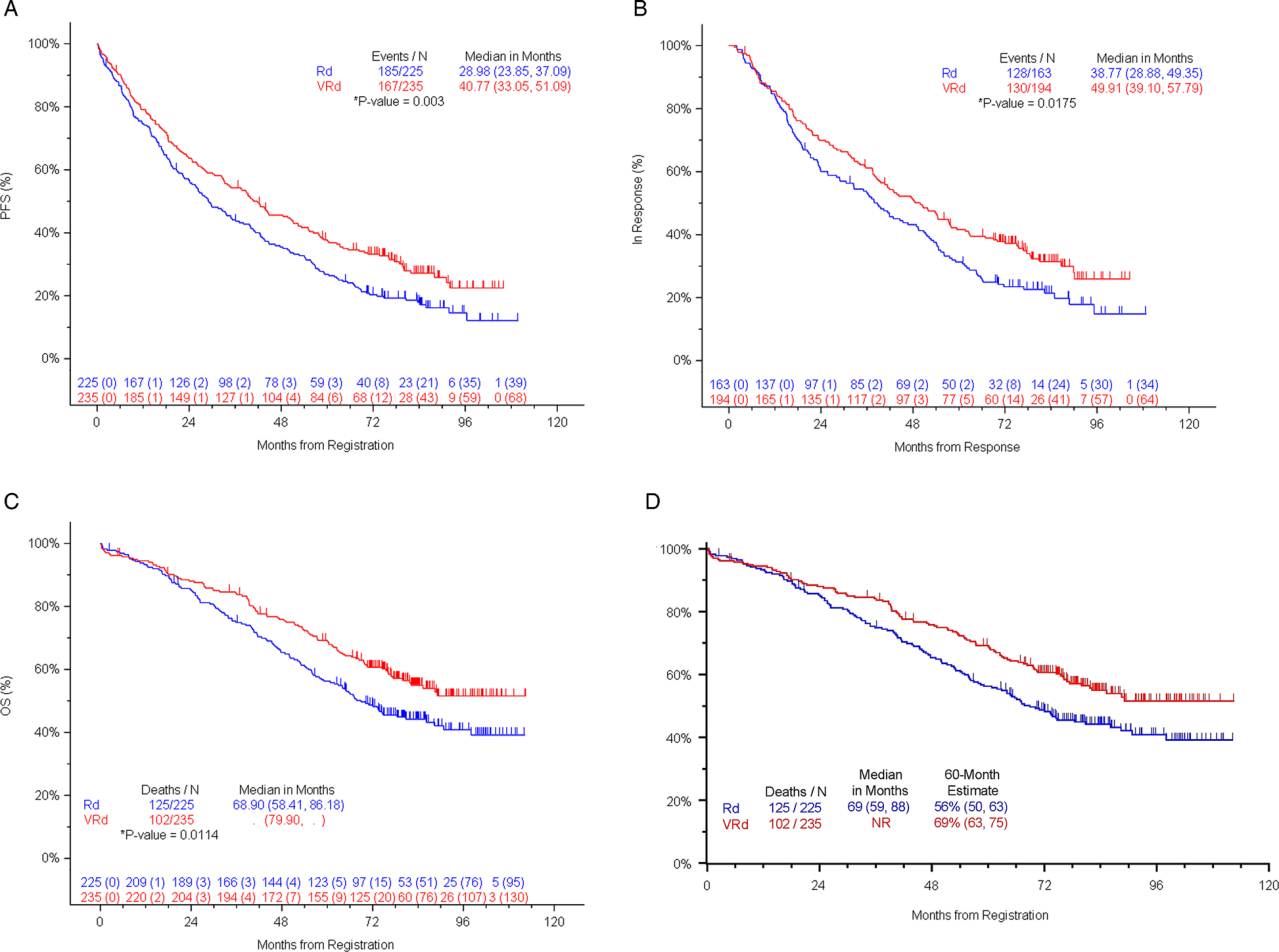
Outcomes for VRd and Rd. **a** Progression-free survival (*N* = 460). **b** Response duration (*N* = 357). **c** Overall survival (*N* = 460). **d** Overall survival (OS) at 5 years.

The median PFS was 41 months for VRd and 29 months for Rd: stratified hazard ratio (96% Wald confidence interval) was 0.742 (0.594, 0.928) and one-sided stratified log-rank *P*-value 0.003 (see Fig. 1a). The response duration was 50 months for VRd versus 39 months for Rd (*P*-value = 0.0175: see Fig. 1b). The median OS for VRd is still not reached with median OS for Rd being 69 months: stratified hazard ratio (95% Wald confidence interval) was 0.709 (0.543, 0.926) and stratified two-sided *P*-value was 0.0114 (see Fig. 1c). The primary report^15^ indicated a median OS of 75 months for VRd. With the update to patient follow-up and events, the estimate for the median is now not yet reached. With longer follow up and updating the median OS for VRd is >84 months. The number of events in the VRd arm changed from 76/242 in the primary report to 102/235 in the current analyses. Because the length of OS for the 133 living patients who received VRd is so much longer at this second analysis, the true median is now not reached. The 5-year estimate for OS was 69% for VRd patients and 56% for Rd patients (Fig. 1d).

Depth of best responses was assessed incorporating new serial data and additional bone marrow results. The rate of overall response rates (ORR) for which non-assessable patients are considered non-responders was 82.9% for VRd versus 72.5% for Rd (*P*-value = 0.006 for response differences using a stratified Cochran–Mantel–Haenszel analysis). A sensitivity analysis including only assessable patients yielded consistent results: the ORR was 90.2% for VRd and 78.8% for Rd and the rate of VGPR or better among assessable patients was 74.9% for VRd and 53.2% for Rd patients (indicated in bold) (see Table 2).

**Table 2:** Confirmed best responses in assessable patients.

	VRd^a^ (*n* = 215)	Rd^a^ (*n* = 207)
Complete response (CR)	24.2% (52)	12.1% (25)
Very good partial response (VGPR)	50.7% (109)	41.1% (85)
**VGPR or better**	**74.9% (161)**	**53.2% (110)**
Partial response (PR)	15.3% (33)	25.6% (53)
**Overall response rate (ORR)**	**90.2% (194)**	**78.8% (163)**
Stable disease (SD)	7.0% (15)	16.4% (34)
PD or Death	2.8% (6)	4.8% (10)

VRd is the bortezomib, lenalidomide, dexamethasone group and Rd is the lenalidomide dexamethasone group.

^a^Both SWOG and IRC stratified Cochran–Mantel–Haenszel analyses indicated improved responses with VRd (odds ratio = 0.528; *P* = 0.006 [ITT] odds ratio = 0.38: *P* = 0.001 [sensitivity analysis].

The impact of treatment within subgroups of interest including intent to transplant, transplant, and age were assessed with age-adjusted multivariable regression techniques (Table 3). In the multivariate regression analysis, the impact of treatment group is retained irrespective of age and intent to transplant classification. These differences are statistically significant for patients <65 years and >75 years (one-sided stratified log rank *P* = 0.0138 and 0.0132, respectively) (Table 4a). The value of VRd was also illustrated by the statistically significantly improved overall survival (versus Rd) for patients <65 years (one-sided stratified log-rank *P* = 0.0138). In addition, Forest plots were performed to assess outcomes for elderly patients. Some results focused on patients for whom there was no intent to transplant and/or no transplant performed are illustrated in Table 4b. In the evaluation of both PFS and OS, there was significant added benefit with VRd versus Rd for patients confirmed to have received no transplant as well as for those for whom there was no intent to transplant and among patients <65 years of age (see stratified hazard ratios and *P*-values, all significant at the <0.03 level).

**Table 3:** Multivariate age-adjusted progression-free survival and overall survival.

	Variable	*n*/*N* (%)	PFS		OS	
			HR (95% CI)	*P*-value	HR (95% CI)	*P*-value
Multivariate	RVd arm	235/460 (51%)	0.77 (0.62, 0.95)	0.013	0.75 (0.58, 0.98)	0.033
	ISS Stage III	155/460 (34%)	1.34 (1.01, 1.77)	0.041	1.98 (1.38, 2.86)	<.001
	ISS Stage II	179/460 (39%)	1.12 (0.86, 1.47)	0.398	1.36 (0.95, 1.97)	0.096
	Intent to Transplant	315/460 (68%)	0.95 (0.74, 1.23)	0.714	0.73 (0.54, 0.99)	0.043
	Age > =65 yr	197/460 (43%)	1.27 (1.00, 1.61)	0.048	1.63 (1.21, 2.19)	0.001

HR—hazard ratio, 95% CI—95% confidence interval, *P*-value from score Chi-square test in Cox regression.

**Table 4:**
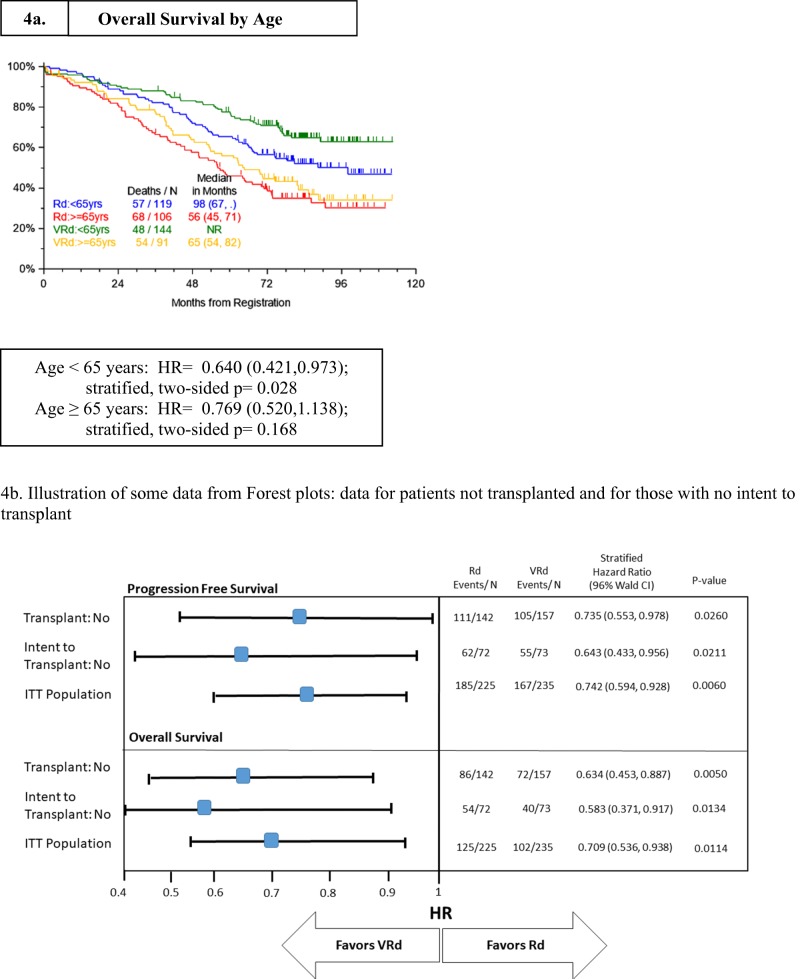
Impact of age on outcomes.

**Fig. 2:**
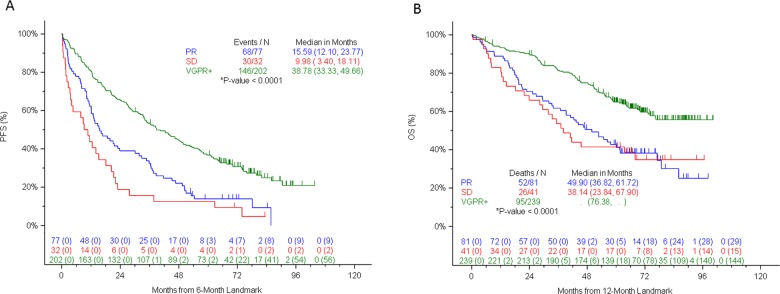
Landmarked outcomes. **a** Progression-free Survival by best response at 6 months. **b** Overall Survival by best response at 12 months.

A number of other parameters were assessed including PFS by best response by 6 months landmarked at 6 months (Fig. 2a) and OS by best response by 12 months from the 12-month landmark (Fig. 2b). The PFS plot in Fig. 2a illustrates the much longer PFS for patients achieving VGPR or better (median of over 38 months versus 10–16 months for other categories: *P* < 0.001). Likewise, the OS is substantially longer at 12 months for patients achieving VGPR or better (median of over 76 months versus 38–50 months for other categories: *P* < 0.0001: Fig. 2b).

Follow-up analyses of outcomes linked to presence or absence of high-risk FiSH abnormalities were conducted (results not shown). Although there were trends towards better PFS and OS with VRd for patients with *t*(4;14) and/or chromosome 17p deletion, differences were not statistically significant primarily because of the limited number of patients with available data. The median duration of lenalidomide plus dexamethasone maintenance was 17.1 months for both arms of the trial. Unfortunately, Time to Next Treatment (TN) was not captured as part of this study.

The treatment emergent adverse events (TEAEs) defined by Common Terminology Criteria (4.0) category and specific toxic effects were fairly well-balanced between the VRd and Rd treatment groups (Table 5). The commonest hematologic adverse events were lymphopenia, thrombocytopenia, anemia, neutropenia, and leukopenia. The commonest non-hematologic adverse events were constitutional symptoms, infection, metabolic and neurologic. The grade 3 or worse neurologic toxic effects were significantly more frequent in the VRd group than the Rd group (34.6% versus 11.3%: *P* < 0.0001) (Table 4). The number of second cancers was 19/235 (8%) with VRd and 16/225 (7%) with Rd. A listing of specific second cancers is provided in Appendix 2.

**Table 5:** Adverse events at least possibly attributable to study drug by category.

Adverse event description	Revlimid/dexamethasone (*N* = 222)					Velcade/Revlimid/dexamethasone (*N* = 234)				
	1	2	3	4	5	1	2	3	4	5
Allergy/immunology	12 (5%)	5 (2%)				10 (4%)	4 (2%)	2 (<1%)		
Auditory/ear	1 (<1%)	16 (7%)				1 (<1%)	8 (3%)			
Blood/bone marrow	22 (10%)	53 (24%)	68 (31%)	39 (18%)		27 (12%)	52 (22%)	70 (30%)	44 (19%)	
Cardiac arrhythmia	5 (2%)	4 (2%)	4 (2%)			10 (4%)	3 (1%)	3 (1%)		
Cardiac general	13 (6%)	9 (4%)	8 (4%)			15 (6%)	17 (7%)	21 (9%)		
Coagulation	1 (<1%)		3 (1%)					5 (2%)		
Constitutional symptoms	61 (27%)	77 (35%)	38 (17%)			60 (26%)	84 (36%)	51 (22%)		
Death					1 (<1%)					2 (<1%)
Dermatology/skin	60 (27%)	23 (10%)	9 (4%)			50 (21%)	41 (18%)	7 (3%)	1 (<1%)	
Endocrine	11 (5%)	8 (4%)				7 (3%)	12 (5%)			
Gastrointestinal	77 (35%)	71 (32%)	19 (9%)			64 (27%)	79 (34%)	51 (22%)	2 (<1%)	1 (<1%)
Hemorrhage/bleeding	13 (6%)	2 (<1%)				9 (4%)	3 (1%)	8 (3%)		
Hepatobiliary/pancreas			2 (<1%)							
Infection	1 (<1%)	31 (14%)	27 (12%)	4 (2%)		1 (<1%)	33 (14%)	34 (15%)	7 (3%)	1 (<1%)
Lymphatics	58 (26%)	19 (9%)	1 (<1%)			73 (31%)	26 (11%)	4 (2%)		
Metabolic/laboratory	56 (25%)	58 (26%)	51 (23%)	13 (6%)		50 (21%)	58 (25%)	57 (24%)	8 (3%)	
Musculoskeletal/soft tissue	25 (11%)	25 (11%)	16 (7%)	1 (<1%)		15 (6%)	31 (13%)	24 (10%)		
Neurology	78 (35%)	44 (20%)	21 (9%)	3 (1%)	1 (<1%)	42 (18%)	70 (30%)	77 (33%)	4 (2%)	
Ocular/visual	21 (9%)	8 (4%)	11 (5%)			39 (17%)	17 (7%)	6 (3%)		
Pain	44 (20%)	29 (13%)	10 (5%)			55 (24%)	43 (18%)	28 (12%)		
Pulmonary/upper respiratory	42 (19%)	27 (12%)	9 (4%)	1 (<1%)		56 (24%)	17 (7%)	15 (6%)	5 (2%)	
Renal/genitourinary	3 (1%)	2 (<1%)	9 (4%)	1 (<1%)		10 (4%)	3 (1%)	6 (3%)		
Secondary malignancy			5 (2%)	1 (<1%)				5 (2%)	2 (<1%)	
Sexual/reproductive function	1 (<1%)	1 (<1%)	1 (<1%)			3 (1%)	1 (<1%)			
Syndromes			2 (<1%)			1 (<1%)	2 (<1%)	4 (2%)		
Vascular		7 (3%)	15 (7%)	6 (3%)		1 (<1%)	9 (4%)	20 (9%)	4 (2%)	

## Discussion

The addition of bortezomib to lenalidomide dexamethasone for induction therapy in previously untreated myeloma results in a statistically significant and clinically meaningful improvement in PFS as well as improved OS with follow-up of 7 years. VRd had an acceptable safety and tolerability profile and continues to represent an appropriate standard of care irrespective of age and transplant intent.

With longer-term follow up, the benefits of VRd over Rd are maintained as in the prior analyses. The PFS benefit is maintained at a median of 41 months for VRd versus 29 months for Rd (one-sided stratified log-rank *P* = 0.003: Fig. 1a). The overall survival benefit is maintained at a median of not yet reached (>84 months) versus 69 months for Rd (one-sided stratified log-rank *P* = 0.014: Fig. 1c). These added benefits with VRd are linked to the deeper responses achieved. With VRd, 74.9% of patients achieved VGPR or better versus 53.2% with Rd (Table 2). The benefits of VRd are also evident in each of the three different age categories, showing a >10-month median benefit across all age groups. Overall survival is also improved both above and below age 65 years. Of particular note, over 55% of patients receiving VRd remain alive at 7 years (median follow-up 84 months).

As part of the report of the primary data^15^, the outcomes benefits with proteasome inhibitor–immunomodulatory drug combinations were fully discussed with reference to prior data^27–30^. It is now remarkable to be able to emphasize that with over 7 years of follow up, the positive impact of the 6 months of VRd induction is retained. This is all the more remarkable in that with VRd induction 34.6% Gd 3 or greater neurologic toxic effects occurred with biweekly intravenous bortezomib. We have proposed that the use of VRd lite and making every effort to maximize the number of cycles of VRd incorporating SQ bortezomib can be reasonably anticipated to further improve outcomes for the maximum number of patients both <65 years and ≥65 years. For the post-induction maintenance, the incorporation of dexamethasone also compromised the ability to continue with ongoing therapy.

As noted, the median duration of maintenance was 17.1 months. The duration is shorter than the 2 years, 2.5 years, and 3 years median values for the studies assessed in the meta-analysis of lenalidomide maintenance after autologous stem cell transplantation in newly diagnosed multiple myeloma^31^. Thus, despite excellent outcomes in the S0777 trial, it is possible that longer maintenance could have provided added benefit.

As noted, the associated rates of second cancers were 8% with VRd and 7% with Rd. Of the 19 and 16 cancers, the numbers of hematologic cancers were 3 for both groups (see Appendix 2 for listing). These percentages and numbers are lower than reported with a median of 79.5 months (versus 84 months for S0777 trial) follow-up in the post-transplant lenalidomide maintenance setting in which the cumulative incidence of second cancers was 5.3% for hematologic plus 5.8% for solid cancers, giving a total of 11.1%. It is worth noting that the S0777 trial included no melphalan and the duration of maintenance was shorter.

While cross-trial comparisons have limitations, it is of interest to compare outcomes achieved with S0777 VRd and Rd regimens with results obtained in the IFM 2009 and MAIA trials^32,33^. In the IFM 2009 trial, patients treated with VRd induction were randomized to receive upfront or delayed ASCT, and the rate of VGPR or better was 77% (versus 74.9% in S0777 trial) while the median PFS duration was 36 months (versus 41 months in the S0777 trial). In the more recent MAIA trial presented as a late-breaking abstract at ASH^33^, and subsequently published^34^, the combination of daratumumab plus Rd was compared with Rd alone in the frontline non-transplant setting. In this study, the VGPR or better rate was 79.3% (slightly higher than with VRd) and the median PFS is not reached but appears likely to be comparable to results with both S0777 and IFM 2009 trials. Two additional points to consider in assessing VRd versus Dara Rd (MAIA regimen) in the frontline setting are the continued use of daratumumab in the maintenance in the MAIA trial (versus no bortezomib in S0777 maintenance) and the uncertain role of Dara Rd in the high-risk setting. As proposed by Kapoor and Rajkumar, head to head comparison of VRd with Dara Rd is required to clarify relative merits^35^. In the MAIA trial, all patients were transplant-ineligible [whereas only some were transplant-ineligible in S0777] and in the IFM 2009 trial, all patients were transplant eligible. Despite these various differences, it is clear that the use of daratumumab can contribute to excellent outcomes in the frontline setting.

The SWOG S0777 trial had several limitations as identified and discussed in the primary report^15^. It is worth re-emphasizing that the use of intravenous bortezomib twice weekly resulted in neuropathy which led to early discontinuation of the VRd induction therapy. Perhaps the most helpful comparison is between the results of the S0777 trial and the recently published Spanish phase 3 PETHEMA/GEM 2012 trial^36^. In the PETHEMA trial, the dose of intensity of bortezomib was reduced to twice per week subcutaneously for 2 weeks out of a 28-day cycle. This resulted in the delivery of all planned six induction cycles, and excellent response and outcomes data including for patients with high risk cytogenetics. A limitation of the S0777 trial was the lack of sufficient cytogenetic data to establish the efficacy for high risk patients.

In conclusion, the addition of bortezomib to lenalidomide dexamethasone for induction therapy in previously untreated myeloma results in a statistically significant and clinically meaningful improvement in PFS as well as better OS with follow up of 7 years. VRd had an acceptable safety and tolerability profile and continues to represent an appropriate standard of care irrespective of age.

## Appendix 1: Trial Profile for VRd=bortezomib with lenalidomide and dexamethasone

*Patients with valid consent and randomized.

## Appendix 2: List of secondary cancers

**Table:**

	VRd	Rd
Hematologic	1. MDS	1. MDS
	2. MDS	2. Myeloid leukemia
	3. Acute Lymphoid Leukemia	3. 2^0^ plasma cell leukemia
Solid	1. Squamous cell carcinoma—neck	1. Neck squamous cell w/ mets
	2. Melanoma	2. NSC lung primary
	3. Prostate	3. Basal cell ca on forehead
	4. R ureter	4. Basal cell carcinoma
	5. Adenoca of lung	5. Basal cell carcinoma
	6. Prostate cancer	6. Squamous cell carcinoma (left cheek)
	7. L upper forehead, L lat cheek	7. Right femur
	8. Lung	8. Left renal cell
	9. Retroperitoneum	9. Uterus
	10, Rectal adenocarcinoma	10. Adenocarcinoma of the lung
	11. Endometrial ca.	11. Prostate
	12. Prostate	12. Pancreatic cancer
	13. Skin, left lateral shin—basal cell carcinoma	13. Melanoma (left lower thigh)
	14. Right shoulder basal cell carcinoma	
	15. Colon adenocarcinoma Stage 1	
	16. Squamous skin	
Total number	19	16
